# Two Sides of a Coin: A Crisis Response Perspective on Tourist Community Participation in a Post-Disaster Environment

**DOI:** 10.3390/ijerph16122073

**Published:** 2019-06-12

**Authors:** Sifeng Nian, Jie Zhang, Honglei Zhang, Jinhe Zhang, Donghe Li, Ke Wu, Xue Chen, Lingling Yang

**Affiliations:** 1 School of Geographic and Oceanographic Sciences, Nanjing University, Nanjing 210023, China; dg1227030@smail.nju.edu.cn (S.N.); zhangjinhe@nju.edu.cn (J.Z.); wuke2018@smail.nju.edu.cn (K.W.); MG1827031@smail.nju.edu.cn (X.C.); mg1827048@smail.nju.edu.cn (L.Y.); 2 School of Business, Anhui University, Hefei 230601, China; adldh@126.com

**Keywords:** crisis response, community participation, community resilience, post-disaster environment, the Wenchuan earthquake, China

## Abstract

This study investigates the attitudes and behavioural intentions of community crisis response and tourism community participation in tourist destinations after the occurrence of a disaster. Further, we built a conceptual model of perceived community participation benefit, community attachment, community resilience, and crisis response and community participation intention and measured it using 556 samples surveyed after the Wenchuan earthquake, China. The results indicated that benefit perception, community attachment, and community resilience have a positive effect on crisis response and community participation. The study further reveals that the local tourism community gradually transforms the negative aspects of terrible disasters into development opportunities. Our case study particularly focuses on the initiative shown by and positive participation of the tourist community in the aforementioned transformation. The study proposes the Attachment, Benefit, and Capability framework of tourist community participation based on the crisis response perspective and expands the boundaries of tourist community participation research. The study has theoretical and practical significance, puts forward relevant countermeasures, and has significant implications.

## 1. Introduction

With the dawn of the new century, the number of disasters and disaster victims and severity of disaster-related economic losses have significantly increased worldwide, and the disaster crisis has become an obstacle to sustainable development. The crisis is defined as a ‘low-probability, high-consequence’ event, that is, a state of emergency in an unpredictable situation [1]. The tourism industry is not immune to crises [2]. The tourism industry is undoubtedly one of the most important components of economies worldwide; however, it is one of the most susceptible and vulnerable components to crises and disasters, as well. In particular, in the twenty-first century, the global tourism industry has been affected by terrorist attacks, political instability, economic recession, ecological security threats, and natural disasters [3,4,5]. However, the management capability and crisis response capacity of the industry to handle complex and critical situations are observed to be limited [6]. In particular, natural disasters such as earthquakes and tsunamis (e.g., the 2008 Wenchuan earthquake and the 2004 Indonesian Tsunami) have significantly affected the local tourism industry. The occurrence of such disasters detrimentally affects tourism development. Natural disasters may destroy the natural environment and resource conditions for tourism development, undermine the service facilities and market order of the tourism industry, and harm tourism practitioners and tourism consumers [7,8]. The livelihood of the community becomes unsustainable, which causes widespread damage to the tourism industry [9,10]. In this manner, disasters form one of the important factors severely restricting the sustainable development of tourism worldwide [11,12,13,14]. 

China is one of the countries that are frequently affected by natural and severe disasters. The Wenchuan earthquake and Southern Snowstorm that occurred in 2008 caused heavy losses to people’s lives and property and, particularly, the development of China’s tourism industry. In particular, the Wenchuan earthquake and its secondary disasters such as mudslides, dammed lakes, and landslides caused heavy losses of life and property to the Chinese people, as well as causing severe losses to the tourism industry in and around the Sichuan Province. On the one hand, disasters frequently occur in the region and, on the other, the local tourism industry is well developed, making the response to and management of sudden crisis events in tourist destinations a time-consuming and difficult task. After the disaster, the tourist destination received both domestic and overseas assistance, particularly through the counterpart assistance program implemented by the central government, which effectively assisted the reconstruction of the destination and recovery of tourism [15]. In addition, following the ancient Chinese survival philosophy of human beings maintaining a harmonious relationship with their land by mastering the laws of nature (人定胜天，simplified Chinese), changing their passiveness and taking action to protect their land, and transforming crises into opportunities, the residents of the tourist destination played a pivotal role in post-disaster land reconstruction and recovery.

The resident of a community is the first person to respond to an emergency crisis; further, since the residents typically are emotionally attached to the place and wish to ensure community livelihood, they are the most direct stakeholders in crisis response, as well. In particular, residents who have experienced natural disasters and have certain capabilities [16,17,18,19] often organize community disaster reconstruction organizations, set up a community crisis response team, and enhance the community’s awareness regarding disaster prevention and reduction [20,21,22]. On the other hand, ‘Misfortune contains a blessing, a blessing hidden scourge’ (祸兮福之所倚 福兮祸之所伏, raised by Lao Zi (about 571 BC–471 BC) was a famous philosopher and Taoist founder in China). From the perspective of tourism development, a sudden crisis may be a ‘double-edged sword’. Although the locals suffer losses, a crisis also brings with it certain ‘opportunities’; sometimes, even a crisis can be turned into an opportunity [9,23]. Holling pointed out that the post-disaster phase is both the most vulnerable period of the region and the best period for local development [24]. Similarly, many scholars emphasize the role of social capital in shaping recovery and resilience in disaster contexts, as well as how the impact can enhance the social capital itself [8,19]. Often, following a disaster, the popularity of surrounding tourist destinations increases; some new tourist landscapes are created; and post-disaster reconstruction involves some new tourism enterprises, launches new tourism projects, and is even further empowered as part of the tourism participation process, which is also the spotlight effect of post-disaster reconstruction [25,26,27,28]. From the perspective of stakeholders of tourism destinations, the research on post-disaster community participation has become a hotspot. Many scholars have examined the factors influencing disaster prevention in the tourist community, including community resilience [29,30,31], community participation support [32,33], place attachment [34,35], community attachment [36,37], community livelihood [38], social capital [39], crisis response [40], and crisis management [4]. Research on tourist communities focuses on community engagement models, community empowerment, community involvement, community attachment, and so on [41,42], and mainly relies on the social exchange theory, local identity theory, and tourism life cycle theory [23,43,44,45]. A review of existing literature based on the perspective of community participation indicates that no previous study has examined community resilience, crisis response, community attachment, and the attitude behaviour theory in an integrated framework using quantitative research methods. This study addresses and focuses on the aforementioned research gap. Further, the current study examines the significant influence of crisis events on the tourism community from the perspective of community participation and crisis response in tourism destinations.

This study explores the mechanism of community participation in tourism based on the perspective of crisis response and constructs a causal model to explain the role and influence of crisis response and community participation after the Wenchuan earthquake. This conceptual model includes perceived the community participation benefit, community resilience, community attachment, community engagement attitude, crisis response attitude, and community participation intentional behavior. In the following sections, the literature review, hypotheses, and conceptual model are presented. Then the questionnaire development, data collection, and analysis are carried out. Finally, the results are discussed and some suggestions and implications are put forward.

## 2. Literature Review and Hypotheses

### 2.1. Perception of the Benefit of Community Participation

Globally, natural disasters have a significant impact on the tourism industry, for instance, earthquakes and tsunamis have caused huge losses to tourist destinations [12], drastically reduced the number of visitors to the relevant locations [13], severely damaged attractions and tourism facilities [11], and made community livelihood difficult to sustain [14]. On the other hand, these crisis events realized some opportunities, as well: They increase global attention on tourist destinations [25], natural disasters often create new tourist landscapes [46], post-disaster reconstruction currently involves some new tourism companies [26] and have recently started some new tourism projects [47]. In particular, collectivist institutions like China adopted counterpart aid construction in areas affected by the Wenchuan earthquake, following which funds were directly injected to 21 provinces and cities, such as Beijing, to support major disaster-affected counties and guide the promotion of post-disaster social and economic development. Further, the post-disaster reconstruction work was carried out smoothly and rapidly, which increased the speed of local social and economic development to reach a certain extent above the pre-earthquake level [15]. In addition, the efficiency and effectiveness of active community participation in post-disaster crisis response mainly depends on the community’s capability to respond well to disasters [48]. While arranging crisis response services and restoring tourism communities, local communities not only receive required material, economic, and policy support but also gain community participation support in tourism development decision-making, tourism management, and employment opportunities. These factors have a positive impact on community involvement and crisis response. From the perspective of the social exchange theory, more benefits of local communities can trigger more community participation support and, thereby, promote community participation, satisfaction, and local community support [23,32,45]. Therefore, we propose the following hypotheses:

**Hypotheses 1** **(H1).** *Community participation benefit has a positive effect on community participation attitude.*

**Hypotheses 2** **(H2).** *Community participation benefit has a positive effect on crisis response attitude.*

### 2.2. Community Attachment

Williams suggests that local attachment consists of local identity and local dependence, in which local dependence is a functional attachment between people and places, whereas local identity is an emotional attachment [49]. A sense of continuity with one’s place should be associated with self-efficacy, that is, a sense of belief in one’s own abilities to meet and manage changing circumstances. Many scholars have further studied the theoretical framework of local attachment and its role in urban disaster prevention and community emotion shaping [34,35,50]. A sense of community facilitates the involvement of individuals in community response following disasters and increases their access to, and utilization of, social support networks coping with a community stressor [51]. Community attachment is a type of local attachment and refers to the extent to and manner in which residents participate in and integrate themselves into the community. Community attachment is a multifaceted psychological process comprising emotions, cognition, and behavioural intentions [52]. It has important emotional functions in facilitating community participation, community support, and crisis response [34]. Community attachment is measured by examining whether an area is an ideal and satisfactory place to live and enables people to feel that they have much in common with the other people in the region and that they fit in this community [53]. Community attachment refers to people’s length of residence in a community and affects their personal well-being or quality of life [36,37]; it is related to the density of established kinship; friendship; and acquaintanceship networks, including one’s feeling of being at home, feeling of sorrow at leaving the community, and interest in their community [54].

The tourism communities’ attachment to the community has promoted the enthusiasm of local communities to participate in tourism development, enhanced the protection of local tourism resources, maintained local cultural initiatives, and promoted the sustainable development of local communities and tourism [55,56,57]. Community attachment is conducive to resolving the contradictions that arise as part of tourism development and encourages the local community to actively respond to sudden crisis events, particularly in response to a catastrophic event. In such an event, due to the limitation of external conditions, the initial dominant force is the community’s self-help, which plays a vital role in reducing the loss of life and property and protecting local cultural customs and the ecological environment [38,58,59]. Studies reveal that community attachment is significantly high and attitudes towards tourism development are very positive in residents who are strongly attached to a place, whereas these aspects are weak in unattached locals [60]. Therefore, we propose the following hypotheses:

**Hypotheses 3** **(H3).** *Community attachment has a positive effect on community participation attitude.*

**Hypotheses 4** **(H4).** *Community attachment has a positive effect on crisis response attitude.*

### 2.3. Community Crisis Response and Resilience

The crisis guidelines for the tourism industry, as specified by the World Tourism Organization in 2003, point out that the so-called tourism crises are those that can shake tourists’ confidence in tourist destinations and affect the latter’s normal operation. Therefore, how to prevent and respond to sudden crisis events in tourist destinations and ensure the sustainable development of tourism has recently become an important research topic in the fields of tourism crisis management and tourism development [8,11,17,31,38]. Scholars mainly study the themes of crisis prevention, crisis response, and recovery of tourism destinations from the perspectives of governmental organizations, local communities, tourism enterprises, tourists, non-governmental organizations, and other tourism destination stakeholders [28,61,62]. As the backbone of tourism development, the local community is both a participant and the guardian of the socio-economic development, cultural heritage preservation, ecological protection, and sustainable development of tourist destinations [59,63]. The active participation of communities in crisis response is very important, since a community is a social unit that has the capacity and resources to activate a response to a disaster and is, hence, called the first responder. Regarding the occurrence of sudden crisis events in tourist destinations and the resultant undermining of normal tourism operations, local communities have greater responsibilities than other stakeholders, since local communities can use their community resources and social capital to better resolve the crises and utilize opportunities. In many cases, the resilience of local communities has enabled the development of tourism communities and rapid recovery from crises and challenges [64,65,66,67].

On the other hand, a tourism community that has experienced major emergency events has the relevant experience and ability to manage a crisis. It has certain social capital and community resilience. Accordingly, the tourism community becomes an important participation force and the first reaction echelon of response to and management of sudden crisis events [68]. Community-based approaches claim to build on existing local knowledge and experience, as well as the resources and coping and adaptive strategies of local residents [69]. Coping capacities are the means by which people or organizations use available resources, skills, and opportunities to overcome adverse consequences that could lead to a disaster, whereas adaptive capacities are the arrangements and processes that enable adjustment through learning, adaptation, and transformation [18,70]. Local responses to disaster are determined by a local community’s coping capacity, adaptability, and community resilience, as well as experience and capabilities such as social support, social participation, and social learning [30,65,66]. Further, the acquisition of crisis response capability may have a positive effect on community participation, and the local community resilience may affect the intention and attitude of the tourism community.

**Hypotheses 5** **(H5).** *Community resilience has a positive effect on community participation attitude.*

**Hypotheses 6** **(H6).** *Community resilience has a positive effect on crisis response attitude.*

### 2.4. Community Participation and Crisis Response Attitude Towards Intentional Behaviour

Due to the importance of developing sustainable tourism, the tourism community extensively participates in research in Western countries. Murphy was the first researcher to introduce community methods in tourism research [71]. Community participation refers to a community’s opinions and needs in the tourism development process, spanning aspects such as policy, development, planning, management, and supervision, and considers it the principal aspect of development participation [42,72]. Community participation enhances community attachment in the process of tourism development, effectively alleviates the contradictions that arise during the development process, protects the ecological environment and social and cultural traditions of the region, and promotes the healthy and sustainable development of tourism destinations [39,41,73]. With the advancement of community participation research, scholars use the social load capacity theory, emotional solidarity theory, social identity theory, social exchange theory, and other theoretical models to perform more systematic research [44]. According to the life cycle theory of tourism, the degree, model, and role of community participation differ for different stages of tourism development. When the community encounters disturbance and destruction caused by sudden crisis events, the role of community participation becomes more distinct [43,74,75].

With respect to crisis management, a community is a region that is inhabited by people within the same scope, consists of a certain population and number of families, and has common disaster risks and disaster reduction goals. The local community has significant social capital, can account for local needs and interests, utilize local resources and potential capabilities more completely, and can compensate for the weak links in community crisis prevention [19,76]. The unique feelings of community attachment formed by local communities are conducive to the protection of local tourism resources, cultural traditions, and ecological environment [20]. An important hospitality function of the tourism community and its tourism industry in a hazardous region is to help prepare for visitors, since tourists are often unfamiliar with the locality and require help [77]. Community members who perceive their lives or livelihoods to be particularly vulnerable to hazards may cooperate more in relevant disaster preparedness initiatives than those who do not [78]. Further, Smith’s research validates the relationship between social capital, place meaning, and perceived resilience [79]. According to the community resilience theory, a community system will be oriented towards community recovery in the case of external power disturbances, which can help the community to effectively cope with sudden crisis events and reduce related harm and loss [27,58]. Vallance used the 2011 New Zealand earthquake as an example to study the community’s response to sudden crisis events and revealed that the community was the ‘first to respond, last to leave’ [59]. Further, Zhao considered the example of approximately 20,000 households that had suffered severe natural disasters in Western China and showed that the participation of local communities positively affect community disaster response and recovery, particularly good social norms, which can accelerate post-disaster recovery and reconstruction [22]. Self-organized efforts by local communities are effective in preparing for crises and ensuring rapid response and faster recovery, implying the importance of informal efforts and suggesting that prior disaster experience can be harnessed in new circumstances [80].

The attitude behaviour theory clarifies that attitude can affect intentional behaviour [81]. The tourism community by itself has some crisis response experience and capability, which influences the attitude towards and nature of community participation [82], similar to how the social exchange theory posits that individual behaviour can be changed to meet certain conditions [45]. A close relationship between the local community’s tourism support, crisis response capability, and community participation attitudes and behaviour can be assumed [23].

**Hypotheses 7** **(H7).** *Community participation attitudes have a positive effect on community participation intentions.*

**Hypotheses 8** **(H8).** *Crisis response attitudes have a positive effect on community participation intentions.*

**Figure 1:**
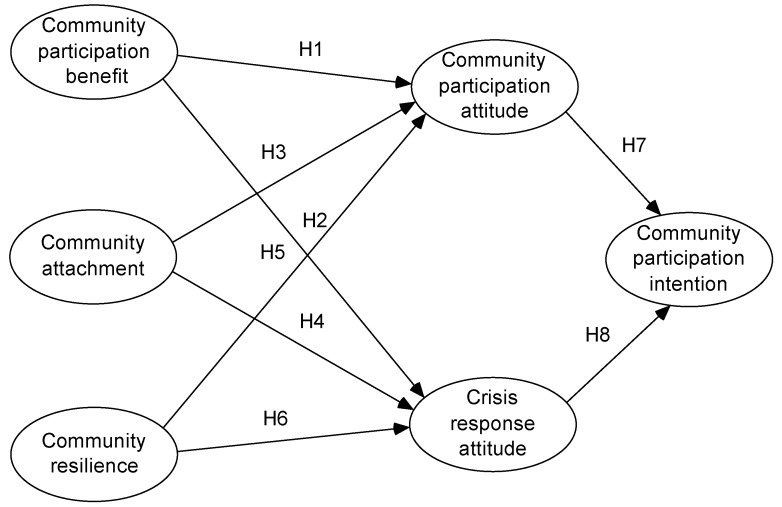
The theoretical model proposed by the study.

The proposed theoretical model is presented in Figure 1.

## 3. Research Design and Methods

### 3.1. Study Area

**Figure 2:**
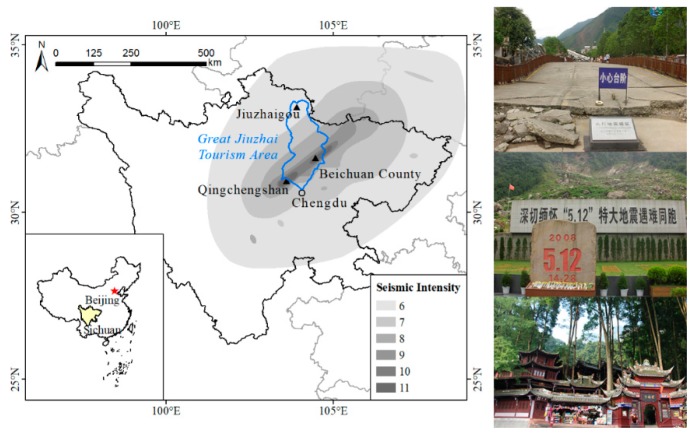
Study area, seismic intensity and panorama.

On 12 May 2008, the 8.0-Richter-magnitude Wenchuan Earthquake occurred in China, killing 69,227 people; injuring 373,643 people; causing 17,923 individuals to go missing; and affecting a total of 46 million persons (Figure 2). Further, it affected an area of 500,000 km^2^ and caused direct economic losses of approximately 845.1 billion yuan. This is the most serious earthquake disaster to have occurred in China over the previous 30 years and the severest natural devastation to occur in the past 10 years. The Sichuan Province, which was the epicentre of the disaster and the pillar of the region’s tourism industry, suffered heavy losses. After the earthquake, the central people’s government in Beijing and the 21 provinces involved in the counterpart assistance program provided disaster relief to the affected county by solving the latter’s basic livelihood problems, assisting the recovery and reconstruction of disaster areas, providing economic support, and receiving positive support and assistance from domestic and foreign individuals, enterprises, and relevant organizations. 

Numerous secondary disasters, such as landslides, mudslides, dammed lakes, and collapses, caused by the Wenchuan earthquake severely affected the Sichuan tourism industry, and several tourism resources, infrastructural components, and service facilities suffered extensive devastation. According to statistics, the Wenchuan earthquake caused a total loss of 46.59 billion Yuan to Sichuan Province tourism, and the number of inbound tourists to the Sichuan Province decreased by 59% in 2008. Domestic and inbound tourists in 2007 were 185.69 million and 1.71 million respectively, but during Wenchuan earthquake in 2008 were 174.56 million and 0.70 million. Following the earthquake, the local government focused on tourism development to boost the community’s morale, promote the development of other industries, and reconstruct the post-earthquake tourism industry. The joint efforts of the entire society, the local government, and the community have enabled the restoration of the local social economy and development of tourism. This study selected the Great Jiuzhai Tourism Area in the Sichuan Province, including the World Heritage Sites Jiuzhaigou, Mount Qingcheng, and the national earthquake relic Beichuan County, as the study area. These tourist destinations are rated 5A, the highest rating level, among scenic spots in China, and which has some typical characteristics and suitability. 

### 3.2. Questionnaire Development

The questionnaire consists of two parts. The first part included the characteristics of community population and tourism participation, including gender, age, monthly income, tourist source, occupation, education, etc. The second part consists of the measurement model scale, including community participation benefit perception [32,33,83], community attachment [60,84], community resilience [34,64], crisis response attitude [40,44], community participation attitude and intentional behavior [14,23], these questions items mainly come from the literature review, and based on preliminary research, comprehensive amendments were designed and developed (The research team has nearly 20 years of experience in the relevant case studies and content). Rating for each statement was based on a Likert scale ranging from (1) strongly disagrees to (5) strongly agrees.

### 3.3. Sampling and Measures

The research team comprised four graduate and four doctoral students, and they participated in questionnaire design and questionnaire distribution training. The questionnaire was designed to reflect the requirements of the local community and was distributed among the Great Jiuzhai Tourism Area’s tourism practitioners. In both the questionnaire and the distribution process, we emphasized how the values of measurements were compared before the earthquake occurrence by including the phrase ‘compared to before the earthquake’ in the questionnaire title. The questionnaire was distributed in August 2012 and later field investigation in October 2014. The research group employed the semi-random sampling method and distributed 590 questionnaires. Among them, 569 were returned and, after excluding incomplete questionnaires, 556 valid questionnaires were obtained; the effective response rate was 94%. At the same time, the researcher’s ideas on the study topic were directly written on the questionnaire to enable a better expression of views.

### 3.4. Data Analysis

This study used confirmatory factor analysis (CFA) by structural equation modelling (SEM). SEM is a method to establish, estimate, and test a causality model, which is widely used in social science research. First, the measurement model was analysed and the model’s validity and reliability were assessed. Second, SEM was used to identify relationships among latent constructs. In this study, the SPSS18.0 statistical software (SPSS Inc., Chicago, IL, USA) was used to process sample data, and both CFA and SEM were conducted using AMOS 17.0 (SPSS Inc., Chicago, IL, USA). When using a structural equation model, one should test whether the model’s parameters have the inverse estimation hypotheses and examine whether the theoretical model has a common method bias.

## 4. Data Analysis and Results

### 4.1. Sample Profile

The demographic characteristics of the sample were as follows (Table 1): The proportion of men (39.9%) and women (60.1%) was approximately coincided with the personnel engaged in tourism services business. Young people under the age of 30 years accounted for 51.6%, and those 30–50 years old accounted for 44.9%. Regarding occupational composition, the percentage of service trade personnel was 46.3%. The education level of participants was mainly middle school (37.6%) and high school (34.1%). The average monthly income was mainly less than RMB 2500, accounting for 83.0%.

### 4.2. Reliability and Validity

The purpose of a reliability test is to assess the reliability, stability, and consistency of the sample data; the greater the reliability, the lower the standard error of the measurement. Testing using the SPSS18.0 software revealed the overall reliability of the scale of Cronbach’s alpha to be 0.935 and the reliability coefficient of each dimension of Cronbach’s alpha to be 0.763–0.888, which is more than the threshold value of 0.7. The Composite Reliability (0.777–0.869) of each dimension was greater than 0.7, which indicated that the model had high reliability. The purpose of the validity test was to investigate the validity of the measurement index in the scale. The Kaiser–Meyer–Olkin (KMO) value of the complete sample testing was 0.933 > 0.9, which showed that the questionnaire had good construct validity. The KMO value of content validity is 0.647–0.866 > 0.6, which indicates that the designed measurement item can represent the content to be measured. The average variance extracted (AVE) values were 0.514–0.689 > 0.5, which indicates that the observed variables can measure the latent variables and the convergent validity of each construct was satisfied (Table 2).

The discriminant validity of the scales used in the study was examined by calculating the AVE and correlations among constructs. This condition occurs when the square root of the AVE is greater than the inter-construct correlation coefficient compared to the correlations for each pair of constructs, as shown in Table 2. In this study, except for pairings such as CR-CPA, CR-CPI, CRA-CPI, and CPA-CPI, other constructs supported the presence of discriminant validity. To further confirm the discriminant validity of these four pairings, a chi-square difference test was conducted [85]. Further, Ping suggests the use of AMOS to test the discriminant validity between two constructs and establish an unconstrained model: First, the variance is set to one (V1 = V2 = 1), although the covariance is not limited, and a constrained model is established. Further, the relevant correlation coefficient between constructs is set to one (both covariance and variance are limited to 1, i.e., V1 = V2 = CO = 1). If the chi-square difference values of the two models do not show a significant difference, it means that there is no discriminant validity between the two constructs, that is, H0: Φ=1 cannot be rejected. If the chi-square difference value, Δχ2, between the two constrained models and the unconstrained model is greater than 3.85 (df = 1, *p* < 0.001), it means that the level is significant and discriminant validity is present [86]. Based on the results shown in Table 3, the chi-square difference values (df = 1, *p* < 0.001) of the four pairing constructs CR-CPA, CR-CPI, CRA-CPI, and CPA-CPI are 92.695, 112.731, 139.703, and 48.367, respectively, and reveal the discriminant validity among the four constructs. In this manner, discriminant validity was confirmed for all constructs. Further, to test the presence of a multicollinearity problem, the variance inflation factor (VIF) was tested. The outcomes showed that all the VIF values were below the threshold of 10, revealing the absence of the multicollinearity problem [87]. Overall, the validity and reliability test results showed that the collected samples are suitable for factor analysis.

### 4.3. Measurement Model

To investigate the relationship between the variables in the measurement model, the structural model was tested and analysed. Accordingly, first, the multivariate normality distribution of samples was tested. The testing revealed the absolute value of observed variables of skewness to be 0.434–1.207, which is less than the threshold value of 2.58. The absolute value of kurtosis was 0.125–3.053, which is less than the threshold value of 10; therefore, the sample data can be considered a multivariate normality distribution. Second, the common method bias testing revealed that bias occurred when the same rater responded to all the questions in the self-administered survey. Hence, we used the Harman’s single factor test for exploratory factor analysis [88]. The first factor explained 18.73% of the total variance, indicating that common method bias was not a serious issue and, hence, could be overlooked. Third, while testing the overall model fit indicators, Hair recommended testing whether there is a violation of the estimation of model parameters, which can proceed from two aspects: Whether there is a negative variance of the error, and whether the standardized parameter coefficient is greater than or equal to one [89]. According to the calculation, the error variance in the model is between 0.013 and 0.058, and there is no negative error variance. The standardized parameter coefficient is between 0.614 and 0.891 and not more than one (Table 4). The results indicating an absence of estimation violation can be tested for model goodness of fit. Finally, we used the maximum likelihood method to estimate the parameters of the theoretical model. Since the relevant fitting parameters were not found to be ideal, we had to further modify the theoretical model [90].

According to the measurement model of SEM, the latent variables were correlated and the relationship between latent variables was established. The correlation coefficients of community benefit, community attachment, and community resilience were 0.62, 0.39, and 0.22, respectively, and became significant at the 0.001 level. The modified structural model fit indices were relatively ideal (Table 5) (chi-square degree of freedom ratio = 3.7, GFI = 0.884, RMSEA = 0.069, IFI = 0.920, TLI = 0.894, CFI = 0.920, PGFI = 0.701, PNFI = 0.770, PCFI = 0.793) [91]. In addition to the revised model, the chi-square degrees of freedom were greater than the ideal value of three, and the remaining indicators could attain the ideal value. According to the study by Nunkoo and Mulaik, when the sample size is greater than 500, the ratio of chi-square degrees of freedom is less than five, but not the usual value of three [90,92]. Therefore, the chi-square degrees of freedom ratio 3.7 is acceptable.

### 4.4. Structural Model

**Figure 3:**
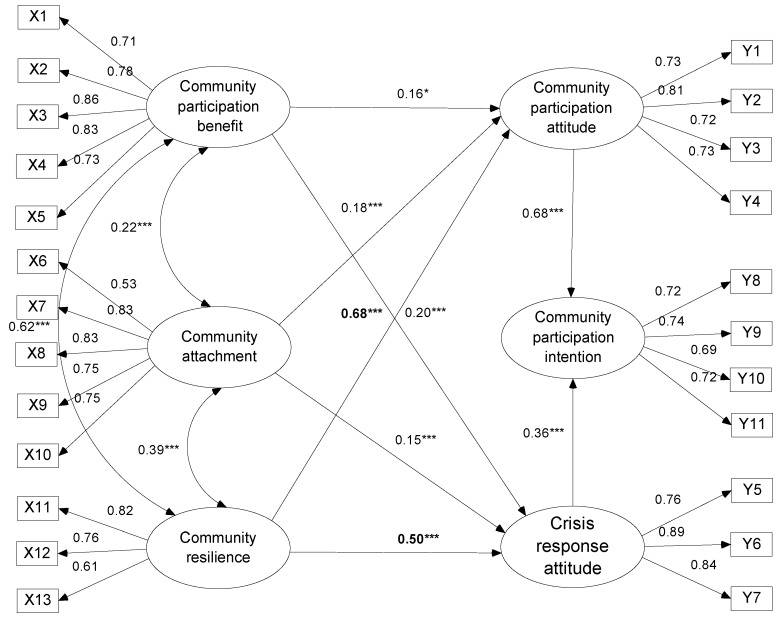
Results of structural model testing. *** Significance at the 0.001 level; * significance at the 0.01 level.

Figure 3 depicts the results of structural model testing and details an overview path of coefficient outcomes.

The results provide sufficient evidence that the theoretical model adequately fits the data and can be tentatively accepted. Figure 3 presents a visual representation of the structural model with standardized coefficients and the significance level of each proposed path. The outcomes indicated that the path coefficients of community benefits, community attachment, and community resilience were statistically significant and consistent with their expected directions; further, they have a positive effect on community participation attitude and crisis response. In particular, the path coefficient of community resilience is greater than that of other coefficients, revealing the community’s capability to respond to tourism crises and play an important role in fighting and adapting to difficult situations. Moreover, the three antecedent dimensions have significant correlations, indicating that benefits, emotions, and abilities interact with and promote each other. The path coefficients of community participation attitude and crisis response are statistically significant and have a positive effect on community participation intention, which indicates that local communities that support tourism development and home reconstruction activities remain active and positive in the face of disaster occurrence.

## 5. Discussion and Implications

### 5.1. Discussion of Results

The study hypotheses were tested, and the outcomes are summarized in Table 6. The results show that all hypotheses are supported. The outcomes showed that tourism community participation and crisis response attitude had a positive influence on intentional behaviour during the period after the Wenchuan earthquake and verified that the local communities’ attitude was positive and active, rather than negative and passive.

The hypotheses H1 and H2 were supported, which was associated with the great concern expressed by the entire society after the earthquake, as well as the strong support provided by the central government and counterpart provinces. However, the tourism industry serves as the vanguard and booster of post-disaster recovery and reconstruction, bringing hope and vitality to the devastated disaster areas. Compared with the pre-earthquake period, the local community’s opportunities in tourism infrastructure, income, employment, skills training, and participation in management decision-making improved after the disaster, and they directly motivated the local community to more actively support the development of local tourism, which is consistent with the conclusions of many relevant studies [23,93,94,95].

The hypotheses H3 and H4 were supported, which indicated that community attachment has a positive effect on crisis response and community involvement. The ‘community attachment’ dimension showed a mean value of 4.2, which further indicated that the local community and tourism practitioners have gained certain support and benefits after experiencing a sudden crisis and have higher local plots and capability to respond to crises. To some extent, the disaster improved the local community’s emotional attachment to the area. Meanwhile, tourism is related to the main livelihood of the local community, and tourism development will promote local image communication, cultural folk heritage preservation, and natural ecological protection. This concern and emotional attachment may have further promoted community participation and crisis response, and they verified the positive effects of emotional and functional factors on post-disaster community recovery and tourism crisis response [34,76,84,96].

The hypotheses H5 and H6 were accepted; they indicated that community resilience has a positive impact on community involvement and crisis response, particularly, the crisis response capability of the post-earthquake community improved. Compared to the pre-earthquake period, the majority of tourism practitioners and community residents were more concerned with crisis response and had more experience in, capability for, and adaptability to responding to risk changes and disaster relief activities after the occurrence of the disaster. These changes have contributed to the effectiveness of post-disaster crisis response and rapid recovery of the tourism livelihoods of locals. After the disaster, governments at all levels and tourism enterprises have increased crisis response and disaster management trainings and drills. The local residents and tourism practitioners pay more attention to disaster prevention and reduction, as well. However, the local community has a wide range of community relationships and social capital, which is conducive to post-disaster recovery and reconstruction. Further, it is demonstrated that the community’s recovery expectations and community attachment improve the locals’ degree of responsiveness to and support for tourism development. Therefore, the initiative of the tourism community is promoted both subjectively and objectively [16,23,64,97].

The hypotheses H7 and H8 were supported, indicating that community participation attitude has a positive effect on community participation intention and is consistent with the attitude behaviour theory and social exchange theory [45,81,94]. Against the background of crisis response, this structural model is rational and representative. It further reveals that the sudden crisis response and community participation in tourism destinations involves a comprehensive system of multi-element interaction [4,33,39]. 

From the perspective of total effect, the value of community participation benefit, community attachment, and community resilience to community participation intention is 0.18, 0.18, and 0.64, respectively, indicating that the local community resilience has a stronger impact on community participation intention. Therefore, we should pay more attention to the community resilience effect when the tourist destination crisis response in the process of disaster recovery.

**Figure 4:**
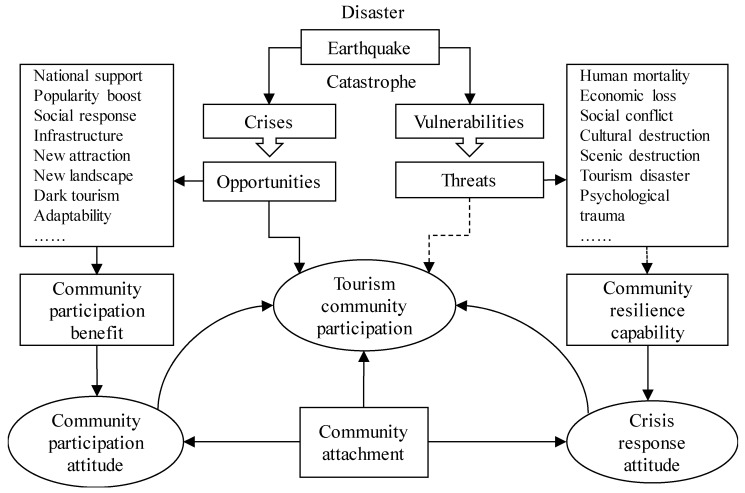
Tourist community participation ABC framework based on crisis response.

In summary, the theoretical framework proposed in the study was verified, and the community participation and crisis response activities remained positive after the earthquake. To a certain extent, some negative factors require further examination and improvement. Based on this discussion, we constructed a tourism community participation ABC framework, including the aspects of community ‘Attachment’, community participation ‘Benefit’, and community resilience ‘Capability’ and exploring the influencing factors and mechanisms of tourism community participation from the crisis response perspective (Figure 4).

### 5.2. Implications

The countermeasures and implications of the study are as follows: First, the local community should understand the key points of community participation and tourism development after a disaster and completely utilize the tourism industry as an opportunity to develop the vanguard. Second, the top-level design plan should be strengthened, a crisis management framework for tourism communities should be constructed, and a long-term mechanism for crisis response in tourism destinations should be established. More focus should be placed on constructing community participation channels, improving tourism participation skills training, increasing community participation empowerment, and further strengthening the breadth and depth of local community participation. Third, the hazard factors should be clarified, negative aspects should be listed, crisis response training and drills should be increased for local communities and tourism enterprises, and the planning and layout of safe evacuation should be strengthened. Fourth, more attention should be paid to benefit sharing in the process of tourism development; the interests of local communities should be considered; community attachments should be utilized, especially in the process of responding to sudden crisis events; and the social capital of local communities and tourism enterprises should be completely mobilised, especially paying more attention to community resilience. These steps provide an inexhaustible driving force for the restoration and reconstruction of the tourism community and the sustainable development of the tourism industry. The Confucian master Dong proposed that the relationship between heaven, earth, and human beings is balanced and harmonious (天生之, 地养之, 人成之); the same philosophy should be applied in disaster reconstruction, enabling human beings to take the initiative in transforming nature, taking advantage of all situations, and overcoming negative predicaments (Zhongshu Dong (BC 179–BC 104), a renowned Confucian scholar in ancient China).

### 5.3. Limitations and Future Research Directions

The limitations and prospects of the study are as follows: First, this study constructs a community participation model from the perspective of crisis response based on positive factors (perceived interests, community attachment, crisis response capability, etc.), which limits the study’s generalizability and comprehensiveness. However, if the research is carried out based on negative factors (perceived costs, psychological trauma, population socioeconomic losses, etc.), one can further expand the scientific and applicable boundaries of this field. Second, one should consider some factors such as the degree of involvement of the tourism industry, degree of crisis impact, and life cycle of a tourism destination, which are not considered in the current study. Third, this survey was performed among tourism practitioners. Therefore, the results obtained may differ from those among general populations. Finally, the case study is limited in that it is based on the Chinese collectivist system and set against the background of a socialist developing country, which may be very different from the non-collectivist system followed by capitalist countries, such as the Western countries. Further, the socio-economic system can have different development backgrounds, which should be considered in future research.

## 6. Conclusions 

This study selected the tourism community following the occurrence of the Wenchuan earthquake in China as the study area and used SEM to examine the tourism community participation mechanism from the crisis response perspective. First, it reveals that the post-earthquake community participation benefit perception, community resilience, and community attachment positively affect crisis response and community participation, and it verifies the effect of attitude on behavioural intention. Second, from the perspective of community resilience, the local community’s social capital, and capability of and experience in managing the crisis, the community attachment complex, and the various support facilities and opportunities gained after the disaster promote the depth and breadth of community participation. This is also an interactive process of mutual promotion and enhancement, rather than being a one-way pre-factor. Third, we proposed the ‘ABC’ framework of tourism community participation based on crisis response, thereby expanding the boundaries of tourist community participation research. This framework has theoretical and practical significance for the enhancement of tourism community participation and tourism crisis management. Finally, this study is based on the community participation theory, involving crisis management theory, community resilience theory, community attachment theory, social exchange theory, and attitude behaviour theory, based on which the research model of tourism community participation and crisis response is integrated. Hence, this study can be considered a comprehensive and pioneering research effort. 

## Figures and Tables

**Table 1:** Sample characteristics.

Items		%	Items		%
Gender	Male	39.9	Tourism revenues accounted for total household income	≤20%	28.3
	Female	60.1		21–40%	22.6
Nationality	Han	61.9		41–60%	22.6
	Zang	19.8		≥61%	26.5
	Qiang	15.3	Profession	Civil servants, managers	10.1
	Other	3.0		Service trade personnel	46.3
Age group	≤20	15.6		Workers, farmers,	14.0
	21–30	36.0		Students	7.9
	31–40	29.5		Other	21.7
	41–50	15.4	Education	Primary school and below	9.0
	≥51	3.5		Middle school	37.6
Income (RMB /month)	≤1000	22.9		High school	34.1
	1001–2500	58.1		College graduate and above	19.3
	2501–4000	10.8			
	4001–6500	4.8			
	≥6501	3.4			

**Table 2:** Reliability, correlation coefficients, and average variance extracted (AVE) results.

Construct	Cronbach’s Alpha	Composite Reliability	AVE	CA	CR	CPB	CRA	CPA	CPI
CA	0.855	0.860	0.56	**0.75**					
CR	0.763	0.777	0.54	0.39	**0.73**				
CPB	0.888	0.887	0.61	0.24	0.63	**0.78**			
CRA	0.862	0.869	0.69	0.39	0.69	0.55	**0.83**		
CPA	0.848	0.837	0.56	0.49	**0.85**	0.61	0.62	**0.75**	
CPI	0.819	0.809	0.51	0.47	**0.81**	0.60	**0.77**	**0.88**	**0.72**

Notes: ^a^ The main diagonal shows the square root of the averaged variance extracted (AVE);. ^b^ Significance, at *p* < 0.05 level is shown in main diagonal, and value in bold is more than 0.70 require further testing for discriminant validity;.^c^ CA, community attachment; CR, community resilience; CPB, community participation benefit; CRA, crisis response attitude; CPA, community participation attitude; and CPI, community participation intention.

**Table 3:** Chi-square difference value testing.

Latent Variables	χ2		**Δχ2**
	The Constrained Model	The Unconstrained Model	(df = 1, *p* < 0.001)
Cov (CR, CPA)	192.497 (df = 14)	99.802 (df = 13)	92.695
Cov (CR, CPI)	270.628 (df = 14)	157.897 (df = 13)	112.731
Cov (CRA, CPI)	334.737 (df = 14)	195.034 (df = 13)	139.703
Cov (CPA, CPI)	230.511 (df = 20).	182.144 (df = 19)	48.367

**Table 4:** Construct dimensions and analysis.

Variables	Mean	SD	Factor Loading
Community participation benefit (CPB)			
X1 Tourism employment opportunities have increased.	3.62	0.92	0.73
X2 Tourism vocational skills training opportunities have increased.	3.56	0.93	0.83
X3 Tourism management opportunities have increased.	3.40	1.00	0.86
X4 Tourism development decision-making opportunities have increased.	3.37	1.01	0.78
X5 Tourism revenues have increased.	3.38	1.03	0.71
Community attachment (CA)			
X6 I pay more attention to local development.	4.25	0.68	0.53
X7 I am already accustomed to living here.	4.18	0.79	0.83
X8 I would like to live here for a long time.	4.29	0.78	0.83
X9 I have a deep affection for this place.	4.29	0.77	0.75
X10 I feel better here than elsewhere.	4.09	0.90	0.75
Community resilience (CR)			
X11 I have improved my capability of coping with tourism crisis	3.83	0.83	0.82
X12 I have improved my capacity to respond to risk changes.	3.76	0.82	0.76
X13 I have improved my adaptability to disaster relief.	3.65	1.06	0.61
Community participation attitude (CPA)			
Y1 I more actively offered bits of advice for tourism development.	3.78	0.88	0.73
Y2 I more actively mobilized friends and relatives to tourism participation.	3.76	0.84	0.81
Y3 I was more concerned about local tourism development.	4.09	0.77	0.73
Y4 I was proud to participate in tourism activities and construct our home.	4.13	0.80	0.73
Crisis response attitude (CRA)			
Y5 My willingness to cherish and protect local tourism resources has been enhanced.	4.04	0.76	0.76
Y6 My willingness to restore and protect natural environment has been enhanced.	4.10	0.75	0.89
Y7 My willingness to rescue and inherit local cultures has been enhanced.	4.09	0.72	0.84
Community participation intention (CPI)			
Y8 My intention to participate in tourism decision-making has been strengthened.	3.67	0.87	0.72
Y9 My intention to engage in tourism-related industries has been strengthened.	3.75	0.84	0.74
Y10 My intention to communicate with visitors has been strengthened.	4.00	0.74	0.69
Y11 My intention to cater to the tourists’ needs has been strengthened.	3.98	0.78	0.72

**Table 5:** Goodness-of-fit indices.

Model-Fit Index	Absolute Index					Comparative Index			Parsimony Index			
	CMIN/DF	GFI	RMR	AGFI	RMSEA	IFI	TLI	CFI		PGFI	PNFI	PCFI
Threshold value	2–5	>0.90	<0.05	>0.90	<0.08	>0.90	>0.90	>0.90		>0.50	>0.50	>0.50
Theoretical model	4.0	0.869	0.049	0.837	0.074	0.908	0.894	0.907		0.698	0.769	0.792
Structural model	3.7	0.884	0.049	0.853	0.069	0.920	0.907	0.920		0.701	0.770	0.793

**Table 6:** Summary of hypotheses testing results.

Hypotheses				SRW	C.R.	Outcomes
H1	Community participation attitude	<---	Community participation benefit	0.16 *	2.97	Accepted
H2	Crisis response attitude	<---	Community participation benefit	0.20 ***	3.51	Accepted
H3	Community participation attitude	<---	Community attachment	0.18 ***	4.59	Accepted
H4	Crisis response attitude	<---	Community attachment	0.15 ***	3.41	Accepted
H5	Community participation attitude	<---	Community resilience	0.68 ***	9.81	Accepted
H6	Crisis response attitude	<---	Community resilience	0.50 ***	7.47	Accepted
H7	Community participation intention	<---	Community participation attitude	0.68 ***	11.25	Accepted
H8	Community participation intention	<---	Crisis response attitude	0.36 ***	7.21	Accepted

Note. *** Significance at the 0.001 level; * significance at the 0.01 level.
